# Circular Agreements—Exploring the Role of Agreements and Deals as a Political Tool for a Circular Economy

**DOI:** 10.1007/s43615-021-00004-5

**Published:** 2021-02-09

**Authors:** Nils Johansson

**Affiliations:** Department of Sustainable Development, Environmental Sciences and Engineering, KTH Royal Institute of Technology, 100 44 Stockholm, Sweden

**Keywords:** Circular economy, Agreements, Deals, Policy, Waste management, Sustainability

## Abstract

A problem for a circular economy, embedded in its policies, tools, technologies and models, is that it is driven by the interests and needs of producers, rather than customers and users. This opinion paper focuses on an alternative form of governance—*agreements*, which thanks to their bargaining approach brings actors from across the value chain into the policy process. The purpose of this opinion paper is to uncover and analyse the potential of such agreements for a circular economy. Circular agreements aim at increasing the circulation of materials and are an emerging form of political governance within the EU. These agreements have different names, involve different actors and govern in different ways. However, circular agreements seem to work when other types of regulations fail to establish circulation. These agreements bring actors together and offer a platform for negotiating how advantages and disadvantages can be redistributed between actors in a way that is more suitable for a circular economy. However, circular agreements are dependent on other policy instruments to work and can generate a free-rider problem with uninvolved actors. The agreements may also become too detailed and long term, which leads to problem shifting and lock-ins, respectively.

## Introducing Circular Agreements

Agreements that regulate legal relations between actors are so essential in industrial symbiosis that they have become a central part of its definition [1]. In industrial symbiosis, agreements establish reliability for waste-based resources by ensuring that the needs of the recipient are met. However, within the larger umbrella of the circular economy, agreements are ignored. Instead, the hope—and thus circular economy research—focuses on business models that shall realise the untapped potential of waste [2]. These models are designed primarily based on the needs and challenges of producers, rather than users and customers [3]. There is thus a need to focus more on the practicalities of the circular economy where the challenges and needs of all actors are included.

There is an understanding among researchers, policy-makers and practitioners that the transition from linear to circular material flows requires political governance [4]. The market failure to include the real social and ecological costs of the linear economy makes circular products typically more expensive than conventional products [5]. At the same time, the waste-based resources may contain higher levels of hazardous substances than the conventional, primary resources [6]. Thus, there are few incentives for customers to use waste-based resources. To make circular solutions more attractive, a variety of policy measures are proposed in the form of deregulation, political objectives, information, procurement, taxes, quotas and other voluntary programmes [7]. Implementing these measures can certainly improve the conditions for a circular economy. However, the suggested measures may rest on a fallacy. These measures create incentives for a circular economy, yet not away from the linear economy [8]. In addition, the proposed policy measures, just like the business models, are primarily designed to improve the conditions for waste producers, rather than the customers who shall utilise the waste-based materials.

The purpose of this opinion paper is therefore to explore an alternative form of governance, *agreements*, which potentially can include the needs of many stakeholders in relation to a circular economy. As previously mentioned, agreements are focused in industrial symbiosis, but primarily between two individual actors. In this opinion paper, agreements are approached as a form of political governance, concluded between collective entities. These types of agreements are emerging in the European Union to increase the level of circulation. But what are *circular agreements* and what potential do they hold for a circular economy?

## Agreements

Agreements can address problems, where other types of governance fail, due to, for example, strong conflicts of interest. This is because agreements are typically a result of a compromise reached through bargaining between the actors involved [9]. Therefore, agreements are standard for regulating, for example, transnational environmental issues such as climate change [10]. However, such international agreements become typically vague and may suffer from the free-rider problem, i.e. actors who ignore the agreement, but may individually benefit from the agreement [11].

This opinion paper focuses on agreements that work on a lower political administrative level and involve practitioners directly affected by the regulation. Such agreements have worked successfully, for example, to substitute legislation in the labour market, between employees and employers [12]. In the Nordic countries, collective agreements have served as an alternative to employment law. Thanks to the agreement, the relationship between the concerned parties has improved, and the local legitimacy and flexibility towards different industries have increased [12]. Implementing agreements means that the role of the actors changes in the policy process. Practitioners are positioned up front in both the negotiation process and the implementation of the agreements, while the state is given a supervisory role to ensure that minimum requirements are met. Agreements as an alternative to legislation entail thus a shift of power from top-down to bottom-up.

### Circular Agreements

The circular economy is typically presented as a utopian green future without any conflicts [13]. Its relation to conflicts is primarily as a problem solver for the number one conflict, between profit and the planet. In practice, however, a circular economy provokes conflicts. For example, those who generate waste want to get rid of it as simply and cheaply as possible. While the potential customers, on the other hand, search for products with as high quality and purity as possible [3]. At the same time, the circular value chains are characterised by high asymmetry in both costs and benefits [14]. The actor who invests most in work, time, and resources and thus bears the costs to upgrade the waste to a resource is rarely the actor who can charge most.

**Fig. 1:**
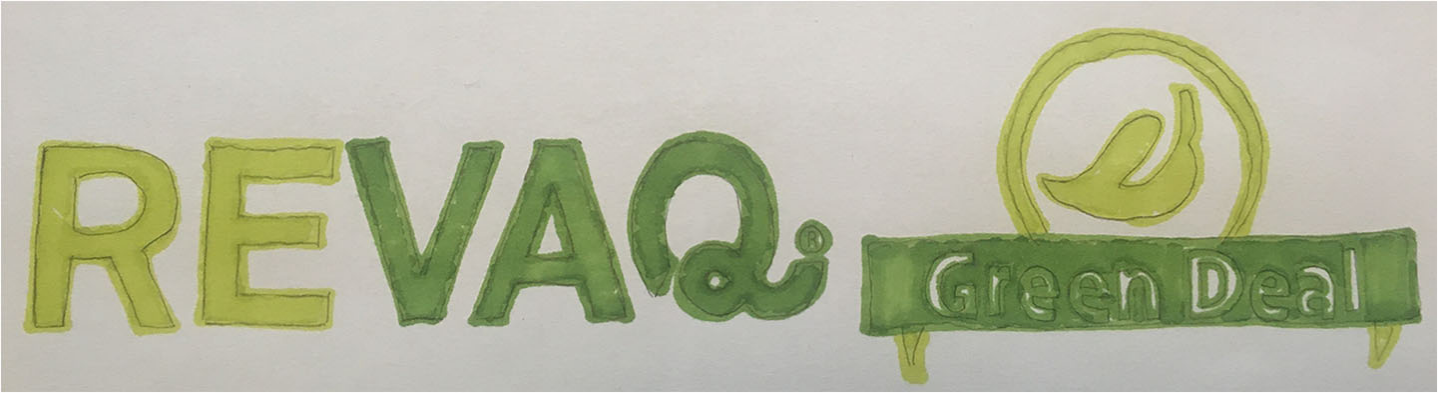
REVAQ and Green Deals are two examples of circular agreements

Circular agreements aim to increase the circulation by addressing the asymmetries and conflicts between stakeholders. It is increasingly being implemented in the EU. For example, *REVAQ* [15] is a Swedish agreement signed in 2009, mainly between the Swedish Water and Wastewater Association (producer) and the Federation of Swedish Farmers LRF (customers) (Fig. 1). The background was an increasing concern that the sewage sludge legislation resulted in unacceptable risks for the farmers. According to the agreement, farmers shall only accept sewage sludge from REVAQ-certified wastewater treatment plants. These plants need to engage in upstream prevention and, for example, disconnect dirty industries from the sewage network. Sewage sludge that shall be applied to farmland needs to meet the concentration limits of 60 hazardous substances and nutrients. The requirements are updated annually through a board that includes all stakeholders in the value chain. Implementation of REVAQ has reduced the presence of hazardous substances in the sludge and increased acceptance of using sewage sludge as a resource [16].

Another example is *Green Deals* in the Netherlands, which are agreed between practitioners and the government. One of the Green Deals concerns the controversial use of incineration bottom ash in the subgrade layer of the road body. Previously, it was legally permitted to use bottom ash with elevated levels of pollution if it was capped under the road. However, this type of application proved to be risky. For this reason, a Green Deal was made between the Dutch government [17] and the waste sector to gradually phase out the use of bottom ash in capped constructions under the road. Since 2020, bottom ash shall primarily be used without insulation, but with much stricter limit values. However, in order to create opportunities for such unrestricted use, the government raised the limits for antimony. It was not considered technically and financially possible to significantly reduce antimony in the ashes. The implementation of this Green Deal has promoted the development of advanced decontamination plants in the Netherlands as well as increased the acceptance of bottom ash as a construction aggregate [18].

## The Potential of Circular Agreements

Circular agreements go under different names. They can be concluded business to business, or between business and the authorities. They can regulate different parts of a material’s life cycle, its origin or intended use as a waste-based resource. However, what these agreements have in common is that the conditions under which a material shall be circulated are stated through negotiation. Thereby, the circular agreements can consider the peculiar complexity of waste [3], for example, the presence of hazardous substances. A closer analysis of these agreements can uncover what the agreements can address in relation to a circular economy, and what they cannot solve.

### The Possibilities of Circular Agreements

#### Stability

In both cases, REVAQ and the Green Deal, the agreements were introduced due to the failure of legislation. For example, REVAQ was initiated when previous regulation was considered insufficient. In such cases, the agreements have offered a flexible and functional alternative to legislation.

#### Inclusion

The negotiation process of the agreements such as REVAQ has included actors across the value chain. This very meeting creates an opportunity to generate trust and understanding between the stakeholders regarding their needs and challenges, which is especially important in waste markets with large uncertainties [19].

#### Reallocation

The agreements have offered an opportunity to negotiate how revenues, costs, risks and responsibilities may be redistributed between actors in a way that is more suitable for a circular economy [14]. For example, the wastewater treatment plants are responsible for the REVAQ-certified sludge after it has left the plant and been applied to the farmers’ land.

#### Predictability

The agreements create predictability by being formulated on a long-term basis. Thanks to the long-term perspective of the Green Deal, the Dutch waste sector invested in innovative technology, infrastructure and knowledge to upgrade the quality of the waste.

#### Standardisation

The agreement is an opportunity for actors to agree on standardisation through stating relevant quality parameters and material properties. As in the REVAQ agreement, standardisation can relate to limit values of both functional resources (e.g. nutrients) and hazardous substances.

### The Problems of Circular Agreements

#### Political Context

The use of bottom ash as well as sewage sludge would be unfeasible without supportive instruments such as landfill bans and taxes [3]. Therefore, accommodating policy instruments that regulate customers’ and producers’ alternative outlets and markets is necessary for the agreements to succeed.

#### Problem Shift

The detailed control of agreements may lead to problem shifting [20]. For example, the Green Deal in the Netherlands makes bottom ashes cleaner and thus more attractive to the market. But, on the other hand, this process requires energy-intensive decontamination processes that may increase emissions of carbon dioxide.

#### Lock-In

Agreements can generate lock-ins [21]. For example, REVAQ has established an acceptable solution to the sludge problem in Sweden, but has hindered innovative solutions such as phosphorus extraction. Moreover, the agreement has caused the authorities to ignore the sludge issue, and the legislation has not been updated since the 1990s.

#### Free-Riders

Membership in industry associations is voluntary, which may create problems for agreements [12]. For example, there are farmers who are not members of the Federation of Swedish Farmers, and do not follow the rules of REVAQ. Instead, they apply sludge in accordance with the outdated legislation from the 1990s. These actors thus avoid the costs of the certificate and increase the local risk of using sewage sludge. However, they may benefit from the outcome of the agreement, such as there being an increased general acceptance of sewage sludge.

#### Evaluation

The effect of agreements depends on what they are related to [22]. For example, the Green Deal in the Netherlands was formulated in order to continue the use of bottom ash. Compared with an alternative situation with no agreement, where the use of bottom ash ceased, the agreement has increased circulation. Compared with history and the situation before the agreement, however, the effect is uncertain as bottom ash was used before the agreement was implemented.

## Conclusion

Agreements offer an inclusive alternative to governing a circular economy, when legislation has failed or has proven insufficient. The potential of circular agreements lies in their ability to bring together and find a compromise between different interests and actors across the value chain. The agreements thereby create the social relations that are necessary for establishing material relations. The specific potential of the agreements lies in their ability to negotiate in detail how the pros and cons of a circular economy shall be distributed across actors. This fine-tuned function makes agreements particularly well suited for handling complex waste streams.

The soft approach of agreements is well adapted to the ongoing liberalisation of the market, where blunt instruments such as legislation are typically undesirable [7]. In addition, agreements are a cheap form of governance, as the responsibility is left to the market. This means that the agreements can be particularly attractive in post-crisis periods such as the time following COVID-19, where the state has a limited budget but great environmental challenges are still alarming. However, circular agreements face a number of challenges that need to be better understood.

The agreements provide an opportunity to bring together different actors, from designers to end users. But, how to trigger the interest to enter into an agreement?

The flat hierarchical structure of the agreements is advantageous for cooperation. However, the lack of hierarchy creates a situation where no one has the authority to intervene. How should compliance be ensured and by whom?

The agreements should be designed in detail to address the complexity of the targeted material. However, this demarcation may lead to problem shifting**.** So on what level of detail should the agreements be formulated?

The agreements should be long term to create stability and predictability, but this may lead to lock-ins. On what time horizon should the agreements be formulated?

The agreements can be an alternative to legislation, but are at the same time dependent on the political context and established policy instruments. So how should the agreements be integrated with current legislation?
